# Stabilizing histamine release in gut mast cells mitigates peripheral and central inflammation after stroke

**DOI:** 10.1186/s12974-023-02887-7

**Published:** 2023-10-07

**Authors:** Maria P. Blasco Conesa, Frank W. Blixt, Pedram Peesh, Romeesa Khan, Janelle Korf, Juneyoung Lee, Gayathri Jagadeesan, Alexander Andersohn, Tushar K. Das, Chunfeng Tan, Claudia Di Gesu, Gabriela Delevati Colpo, Jose Félix Moruno-Manchón, Louise D. McCullough, Robert Bryan, Bhanu P. Ganesh

**Affiliations:** 1 Department of Neurology, The University of Texas McGovern Medical School, Houston, TX 77030 USA; 2 Department of Anesthesiology, Baylor College of Medicine, Houston, TX USA

**Keywords:** Histamine, Mast cells, Stabilizer, Gut–brain axis, Neuroinflammation, Cytokines, Peripheral factors, Microbiome

## Abstract

Stroke is the most common cause of long-term disability and places a high economic burden on the global healthcare system. Functional outcomes from stroke are largely determined by the extent of ischemic injury, however, there is growing recognition that systemic inflammatory responses also contribute to outcomes. Mast cells (MCs) rapidly respond to injury and release histamine (HA), a pro-inflammatory neurotransmitter that enhances inflammation. The gut serves as a major reservoir of HA. We hypothesized that cromolyn, a mast cell stabilizer that prevents the release of inflammatory mediators, would decrease peripheral and central inflammation, reduce MC trafficking to the brain, and improve stroke outcomes. We used the transient middle cerebral artery occlusion (MCAO) model of ischemic stroke in aged (18 mo) male mice to investigate the role of MC in neuroinflammation post-stroke. After MCAO we treated mice with 25 mg/kg body weight of cromolyn (MC stabilizer) by oral gavage. Cromolyn was administered at 3 h, 10 h, 24 h and every 24 h for 3 days post-stroke. Three control groups were used. One group underwent a sham surgery and was treated with cromolyn, one received sham surgery with PBS vehicle and the third underwent MCAO with PBS vehicle. Mice were euthanized at 24 h and 3 days post-stroke. Cromolyn administration significantly reduced MC numbers in the brain at both 24 h and 3 days post-stroke. Infarct volume was not significantly different between groups, however improved functional outcomes were seen at 3 days post-stroke in mice that received cromolyn. Treatment with cromolyn reduced plasma histamine and IL-6 levels in both the 24-h and 3-day cohorts. Gut MCs numbers were significantly reduced after cromolyn treatment at 24 h and 3 days after stroke. To determine if MC trafficking from the gut to the brain occurred after injury, GFP^+^MCs were adoptively transferred to c-kit^−/−^ MC knock-out animals prior to MCAO. 24 h after stroke, elevated MC recruitment was seen in the ischemic brain. Preventing MC histamine release by cromolyn improved gut barrier integrity and an improvement in stroke-induced dysbiosis was seen with treatment. Our results show that preventing MC histamine release possesses prevents post-stroke neuroinflammation and improves neurological and functional outcomes.

**Graphical abstract:**

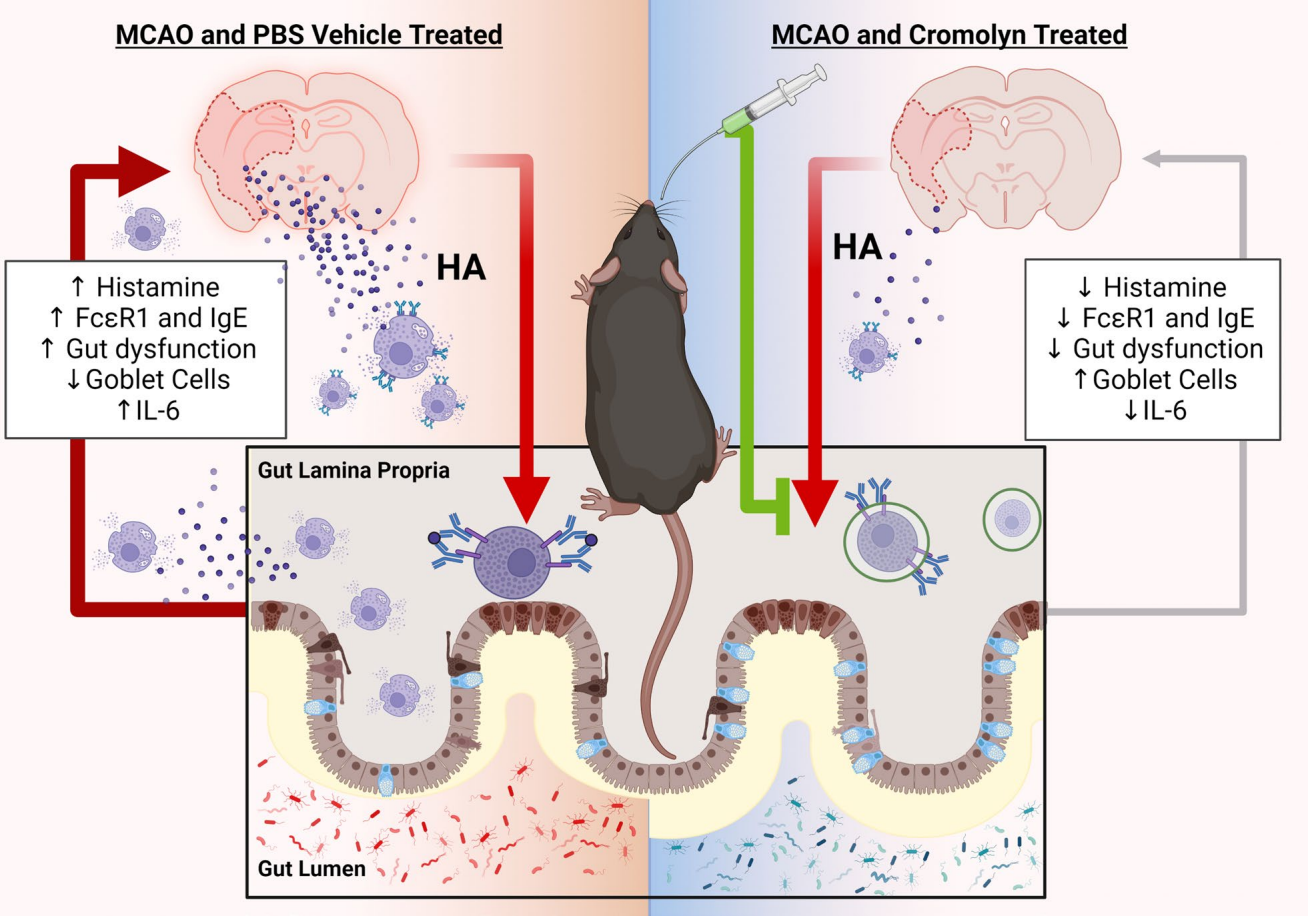

**Supplementary Information:**

The online version contains supplementary material available at 10.1186/s12974-023-02887-7.

## Introduction

The importance of the bidirectional communication within the “gut–brain axis” in response to stroke is increasingly recognized [1–3]. Many systemic inflammatory processes are activated by ischemic stroke and contribute to brain injury and mortality [4–6] especially in the elderly. In the U.S., over 10% of patients die 1 month post-stroke [7], and survivors go on to suffer an accelerated decline in function, cognition, and quality of life [8]. Increasing evidence suggests that peripheral inflammatory responses after stroke play an important role in determining neurological outcomes [9]. Mast cells (MCs) are of hemopoietic origin, are distributed in most tissues, and are found predominantly in the lung and gut mucosa. MCs serve as primary immune sentinel cells to respond against injury and invading pathogens and environmental antigens/allergens [10]. MCs release both pre-stored and newly synthesized mediators, specifically histamine (HA) [11–17]. HA is a pro-inflammatory neurotransmitter that enhances inflammation [18–21], and is required for the maturation of MC progenitors [15]. Mucosal MC progenitors constitutively home to the intestinal mucosa and are recruited and mature under inflammatory conditions [22, 23]. Prior studies have shown the involvement of MCs in stroke outcomes, but their source is not well understood [3], one of the major sources of HA releasing MCs [24, 25]. We hypothesized that gut MCs are a major source of the systemic rise in pro-inflammatory histamine after stroke. Stroke elicits a vicious cycle of peripheral inflammation through communication within the brain–gut axis (BGA). This leads to severe gut dysregulation, which is worse in aged animals [26–28]. We determined if stroke in aged animals leads to gut mucosal MC activation, if stabilization of gut MCs decreased peripheral and central inflammation and reduced MC trafficking to the brain leading to improved post-stroke outcomes.

Prior studies have shown that gut MCs are increased after stroke with a corresponding elevation in histamine levels in both brain and blood plasma as early as 6 h post-stroke. Histamine levels were significantly elevated and increased pro-inflammatory cytokines were seen in aged animals compared to young animals at 24 h post-stroke [3]. However, it is unknown if directly stabilizing MCs could prevent histamine release and progenitor MC maturation would have a positive effect on preventing post-stroke neuroinflammation. To investigate the hypothesis, we treated aged WT mice with cromolyn (a mast cell stabilizer) at 3, 10 and every 24 h for up to 3 days (d) after middle cerebral artery occlusion (MCAO). We examined MCs in the brain of human stroke patients, investigated MCs in mice after stroke by flow cytometry, administered a MC stabilizer to aged mice and measured plasma histamine, MCs and cytokines in mice and stroke patients. To directly determine if MC traffic to the ischemic brain, we used adoptive transfer of GFP-labeled MC. Overall, our data showed that oral administration of cromolyn, an MC stabilizer, reduces circulatory histamine levels and prevent inflammatory signals from the periphery to the brain and reduces neurological deficits and improves outcomes.

## Results

### Mast cell infiltration was significantly elevated at the acute and sub-acute phase of stroke in human brains

**Fig. 1:**
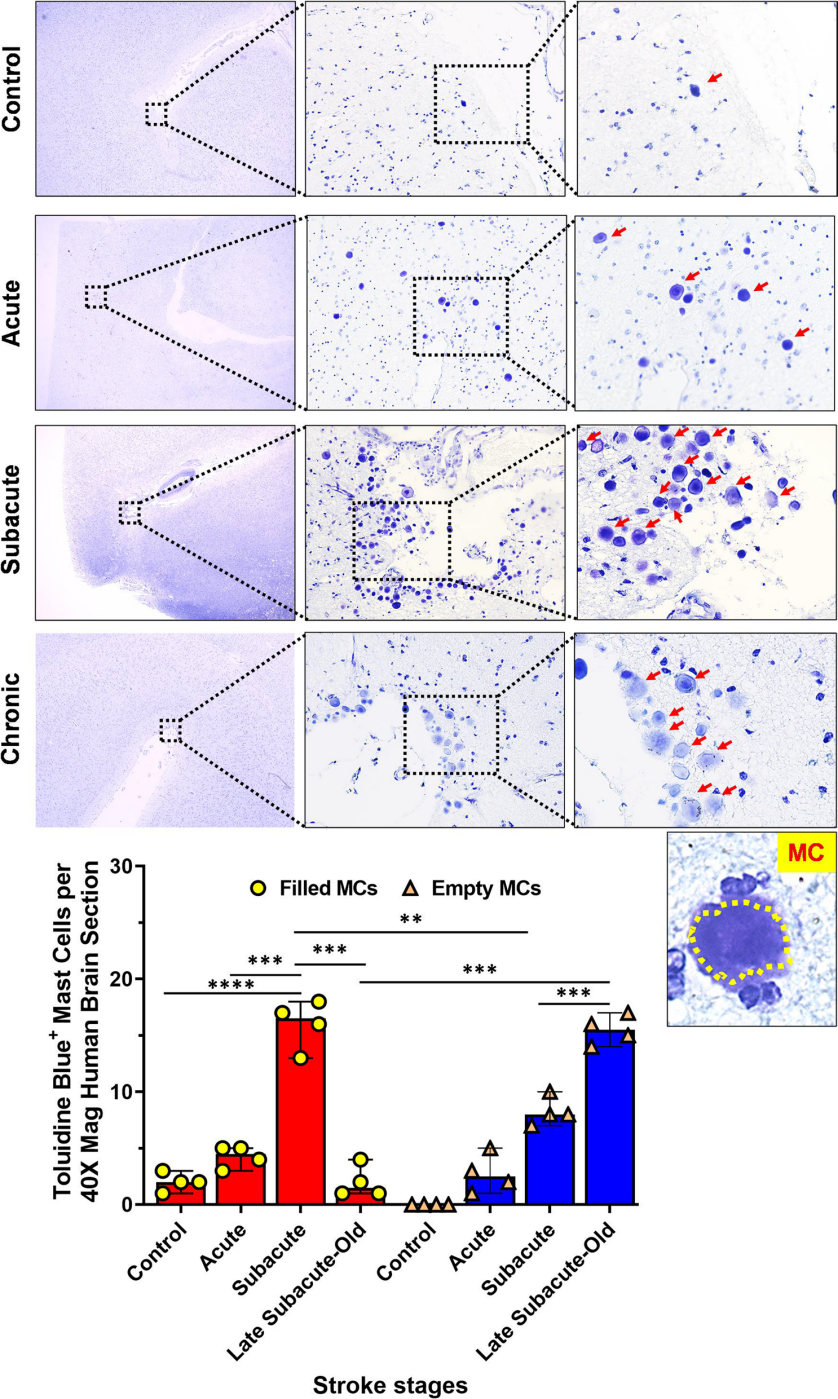
Mast cells in human infarct region of samples obtained by acute, sub-acute and chronic (late) timepoints: MCs analyzed and quantified by Toluidine Blue (TB) staining in human brain samples. **A** Acute, sub-acute and later sub-acute (old) stages, with MC marked with red arrows. Mast cells (histamine filled) stains in purple (TB staining). Magnification; 20X. **B** Quantification of MCs shows an increase in MC infiltration during the acute phase and significant increase of MCs at the sub-acute phase. At this phase, the histamine-filled MCs are higher compared to emptied MCs. The late sub-acute phase shows an increase in the empty MCs. Data are expressed as mean ± SEM, as well as individual values. Two-way ANOVA and Turkey’s multiple comparison show the statistical significance among the groups (*n = *4 per group). *P < 0.05, **P < 0.01, ***P < 0.001, ****P < 0.0001

To investigate the importance of mast cells one of the primary responders after injury [10], we examined MCs in human brain samples from acute, sub-acute and chronic stroke patients. We examined both sexes with appropriate age-matched controls. Using toluidine blue staining, we found an increase in MC infiltration in the brain during the acute phase of stroke (Fig. 1). A significant increase in MCs was also seen in the sub-acute phase of stroke and an increase in histamine-filled MCs compared to empty MCs was seen. Interestingly, we also found increased MCs at the chronic phase of stroke in the human brain. However, most of the MCs found in the late sub-acute and chronic stroke samples had MC that were empty of histamine. This suggests that MC activation occurs acutely after stroke, potentially increasing inflammation by histamine release at the site of injury (Fig. 1). To confirm the importance of MC histamine release, we then treated mice with cromolyn to and examined infiltration of MC and HA release in the brain after stroke [22, 23].

### Early cromolyn treatment significantly reduced neurological deficits at 24 h and 3 days after stroke

MC’s are elevated in the gut after stroke, accompanied by elevation in histamine levels in both the plasma and the brain 24 h after stroke in aged mice. Human autopsy brain samples demonstrated that histamine-filled MCs were elevated at the acute to sub-acute phase (Fig. 1), we then investigated the importance of MC-specific HA release using animal models. We used cromolyn treatment, a MC stabilizer, to prevent HA release from MCs after stroke. We determined the effect of the cromolyn treatment (at 3-, 10- and at 24- h) on stroke outcome and assessed behavioral deficits after stroke in aged mice at 3 and 24 h post-stroke. MCAO and sham groups were treated with cromolyn or vehicle (PBS) by oral gavage. Mice were assessed using the NDS scale at 3 h (pre-treatment) and 24 h (post-treatment) by an investigator blinded to treatment group.

**Fig. 2:**
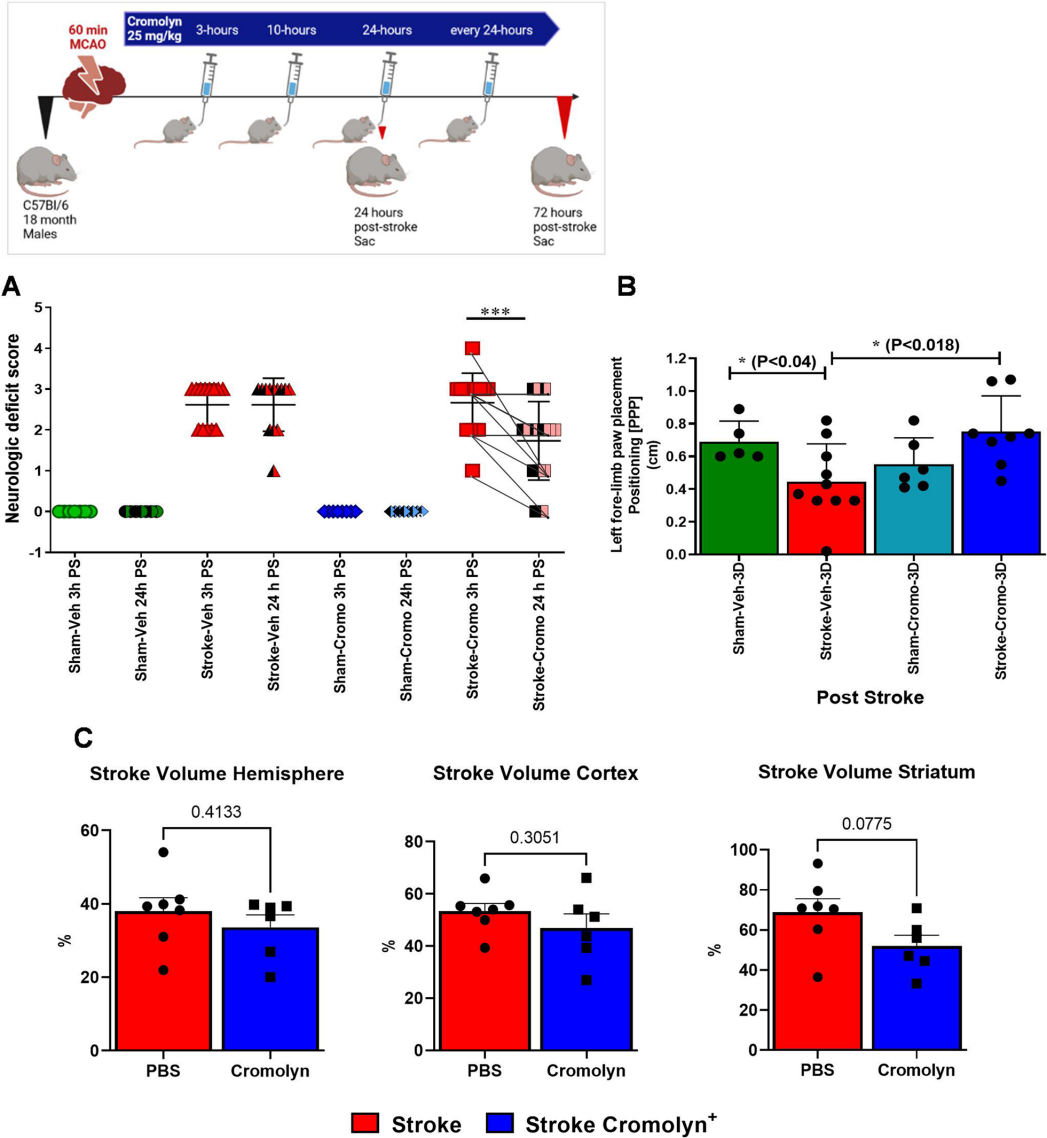
**A** Neurologic deficit score (NDS) at 3 h and 24 h after treatment in sham and stroke aged mice with cromolyn or vehicle treatment shows significant improvement in post-stroke recovery and outcomes. Two-way ANOVA and Turkey’s multiple comparison show the statistical significance among the groups (*n = *12–15 per group). h—hours, PS—post-stroke, crom—cromolyn. **B** Digigait paw positioning at 3 days PS. The motility activity is significantly improved 3 days after stroke in the cromolyn-treated mice compared to the vehicle-treated mice (*n = *5–10). **C** Relative infarct volume 3 days after stroke in cromolyn-treated and PBS-treated mice. Cromolyn-treated mice had no significant changes in infarct volume in both cortex and striatum compared to the PBS group (striatum showed a trend in reduced infarct volume, *n = *6–7, *t *Student analysis). Data are expressed as mean ± SEM, as well as individual values, and are obtained from > 4 independent experiments at various times. *P < 0.05, **P < 0.01, ***P < 0.001

Mice that underwent MCAO had a significantly higher NDS score than sham mice. There was no difference in NDS score between cromolyn and vehicle-treated mice 3 h after stroke. However, at 24 h we saw a significant reduction in the NDS score between the groups (*P* < 0.001), and the cromolyn-treated group had improved functional recovery with cromolyn administration at 3 h, 10 h, and 24 h (Fig. 2A). Somewhat surprisingly, cromolyn administration did not significantly reduce infarct volume especially in the cortex (Fig. 2C), suggesting the beneficial effects of cromolyn were independent of an acute neuroprotective effect. In addition, we found significant improvement of forelimb paw positioning in aged animals after cromolyn treatment (at 3 h, 10 h, every 24 h up to 3 d) compared to vehicle-treated MCAO groups at 3 days after stroke in digigait testing (Fig. 2B). No significant differences were seen in global motor function in the open field test; no difference in depressive phenotypes was seen in the tail suspension test (Additional file 1: Figure S1). Overall, cromolyn treatment improved NDS score and improved stroke deficits as early as 24 h after stroke.

### Brain MCs infiltration was significantly reduced after cromolyn treatment 24 h and 3 days after stroke

**Fig. 3:**
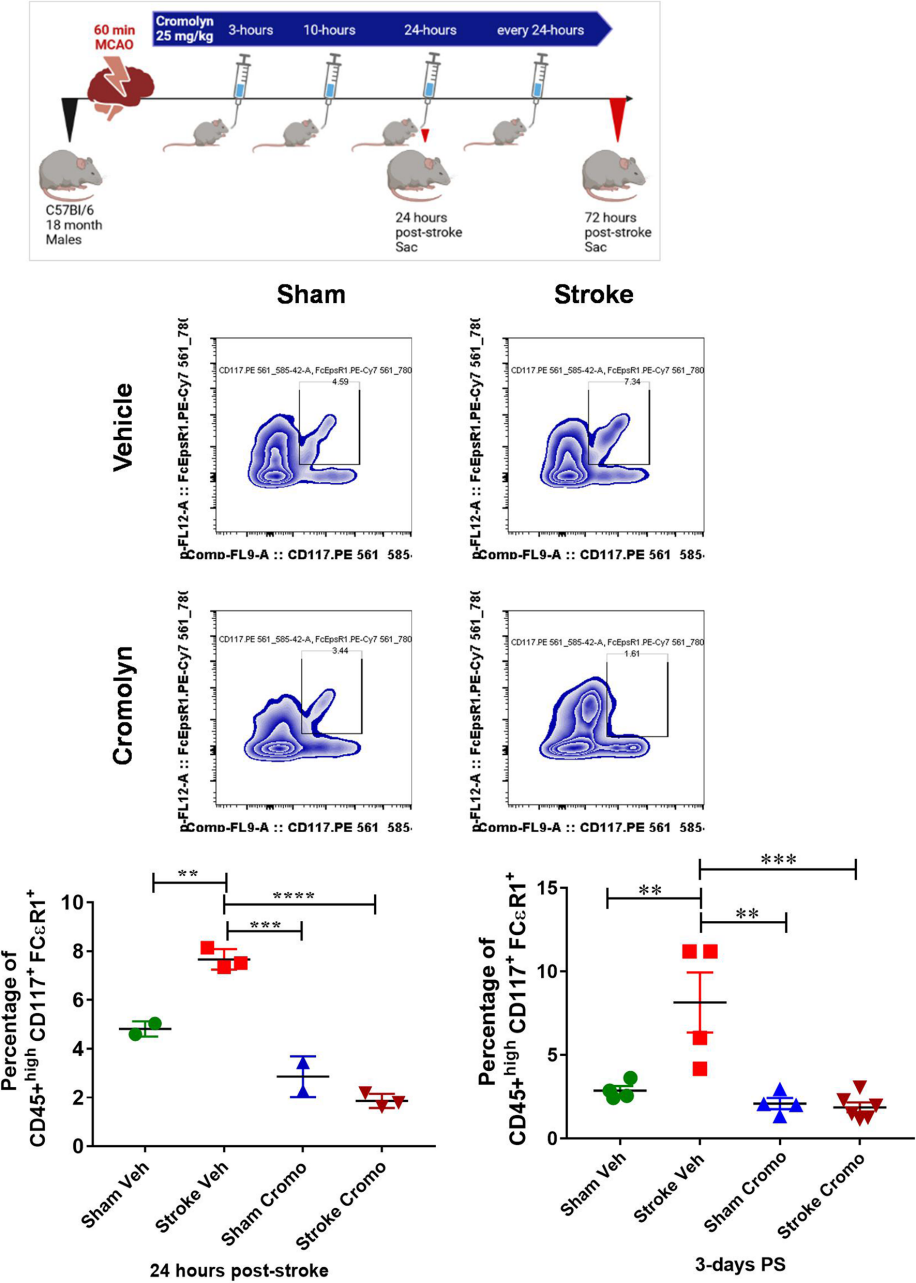
Flow cytometry analysis on the brain tissue revealed that there is a significant increase of MC infiltration in the brain of post-stroke mice compare to sham mice. Importantly, administration of cromolyn orally as early as 3 h, 10 h and 24 h caused a decrease in MC numbers in the brain post-stroke compared to the vehicle treatment groups at 24 h and 3 days after stroke. Two-way ANOVA and Turkey’s multiple comparison show the statistical significance among the groups. Data are expressed as mean ± SEM, as well as individual values, and are obtained from three independent experiments at different time points (*n = *4–6). *P < 0.05, **P < 0.01, ***P < 0.001.

We investigated if oral cromolyn treatment (at 3 h, 10 h and every 24 h post-stroke) had an impact on MC recruitment at 24 h and 3 days after stroke in the ischemic hemisphere of aged WT mice using flow cytometry (Fig. 3). After exclusion of B cells, T cells, macrophages, and dendritic cells, MCs were identified as CD45 + ^high^ FCεR1 + CD117/c-Kit + population. Aging leads to elevated MCs in the gut and brain [3]. We found that MCAO in aged mice leads to a significant elevation in MCs infiltration in the infarct region at 24 h (*P* < 0.01) that persisted through 3 days after stroke compared to age-matched sham groups. However, cromolyn treatment initiated at 3 h after stroke with repeated dosing at 10 h and every 24 h after stroke led to a significant reduction (*P* < 0.0001) in MCs recruitment to the infarct area and this effect was also seen at 3 days post-stroke (*P* < 0.001) compared to vehicle-treated MCAO mice. Overall, this suggests that stroke initiates the recruitment of MCs from the periphery to the ischemic brain in aged mice, and this can be attenuated with administration of cromolyn.

### Pro-inflammatory cytokines were upregulated after acute stroke in both mice and humans

**Fig. 4:**
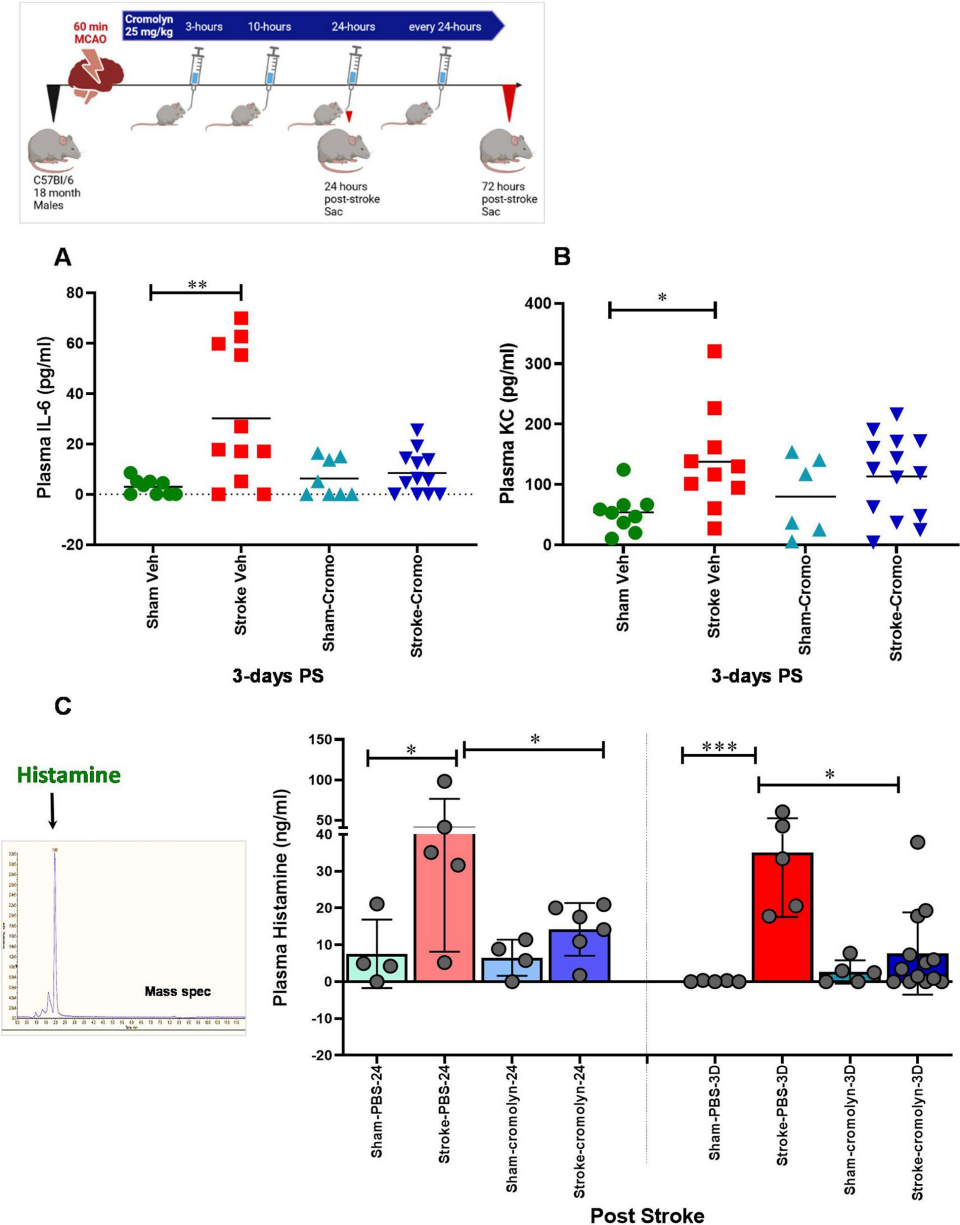
**A** Mass spectrometry measurement of circulating histamine in the blood plasma of post-stroke animals with and without cromolyn treatment. Plasma levels of histamine at 24 h and 3 days post-stroke were significantly reduced after the cromolyn treatment. Data are expressed as median ± range, as well as individual values. **B** Increased levels of plasma IL-6 and **C** KC at 3 days after stroke when compared to age-matched shams. Cromolyn administration significantly downregulated both IL-6 and CXCL1 levels in the blood plasma post-stroke. Data are expressed as mean ± SEM as well as individual values, and are obtained from > 4 independent experiments at various times. Two-way ANOVA and Turkey’s multiple comparison show the statistical significance among the groups. *P < 0.05, **P < 0.01, ***P < 0.001

Interleukin-6 (IL-6) is a key pro-inflammatory molecule and mediates stroke-induced inflammation [29–31]. IL-6 increases MCs maturation [32] by inducing the FcεR1 receptor on MCs [33–36] and upregulates HA production. We found significantly increased levels of plasma IL-6 at 3 days (*P* < 0.01) after stroke in aged mice compared to age-matched shams (Fig. 4A). Similarly, KC (CXCL1, a chemoattractant factor for neutrophils) levels were significantly elevated in the blood plasma 3 days (*P* < 0.05) after stroke compared to sham controls (Fig. 4B). Neutrophil infiltration into the injury site is associated with poorer outcomes and elevations in neuroinflammation after stroke. Interestingly, cromolyn treatment led to a significant downregulation of both IL-6 and CXCL1 levels in the blood plasma post-stroke.

Interestingly, human blood plasma samples obtained from stroke patients also showed upregulation of IL-6 levels confirmed at 12 h after stroke, which was also seen in the blood plasma obtained from patients at 24 and 72 h post-stroke. Similarly, granulocyte colony stimulating factor (G-CSF) measured in human blood plasma was significantly elevated 12 h after stroke and the levels remained elevated at 24 and 72 h after stroke compared to age-matched healthy human controls (Additional file 2: Figure S2).

### Cromolyn treatment reduced histamine levels in the plasma of aged animals at 24 h and 3 days post-stroke

HA is released by MCs as an immediate response to tissue damage and initiates an inflammatory response [11–17]. Aging is associated with increased HA levels in the plasma and the brain after acute stroke [3]. Therefore, we tested the effect of the mast cell stabilizer, cromolyn on the HA levels in the blood plasma at 24 h and 3 days post-stroke. Our results show that aged mice exhibit a significant increase in plasma HA levels at 24 h (*P* < 0.05) and 3 days after stroke (*P* < 0.001) compared to age-matched shams. The administration of cromolyn (at 3 h, 10 h and every 24 h post-stroke) significantly reduced HA levels in the blood plasma measured at 24 h (*P* < 0.05) and at 3 days (*P* < 0.05) after stroke compared to the vehicle-treated mice (Fig. 4C).

### Cromolyn treatment reduced the MC population and elevated muc2 goblet cells within the gut mucosa after stroke

**Fig. 5:**
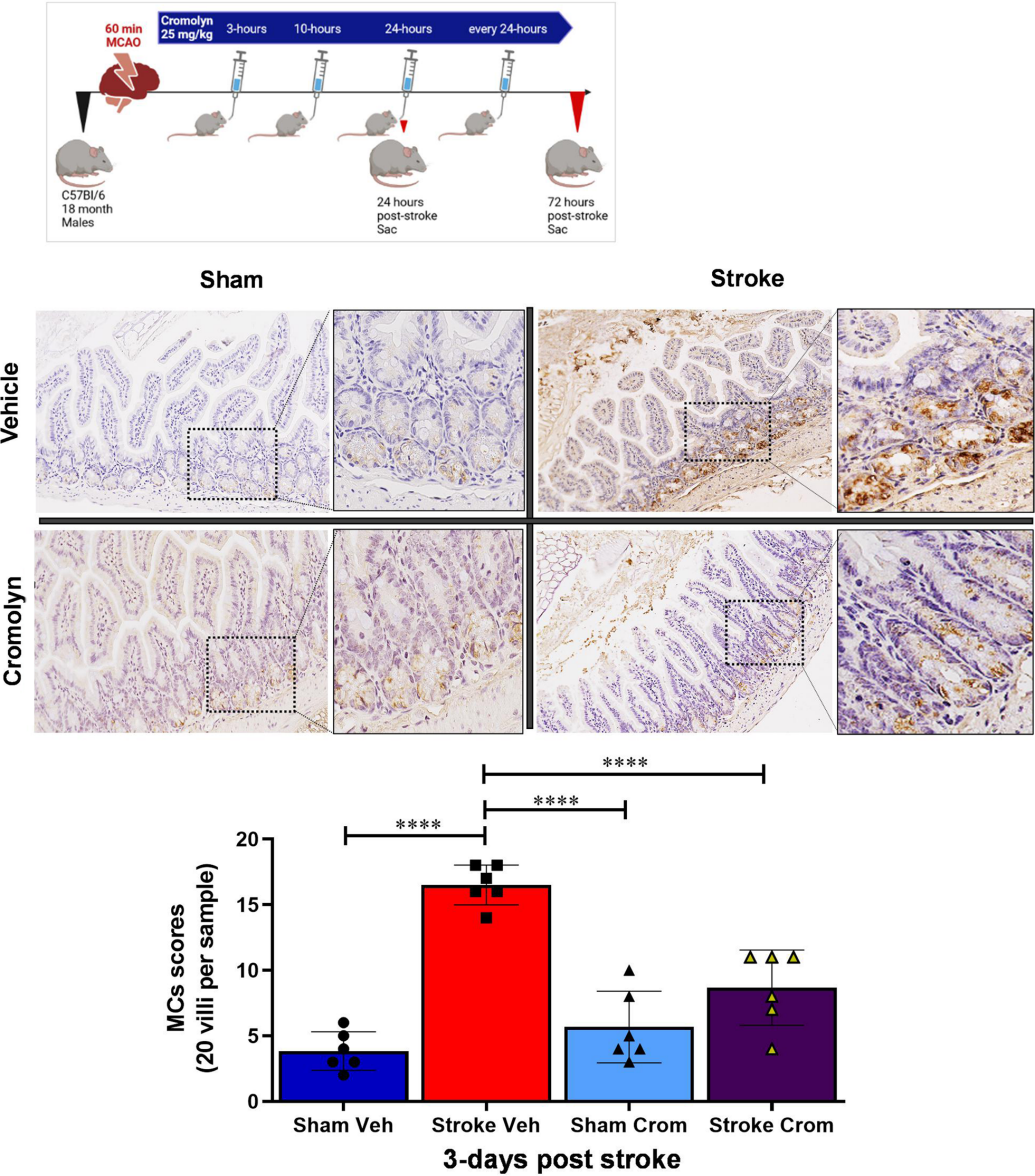
Immunohistochemistry staining followed by quantification of mast cells in the ileal tissue of mice. Significant reduction in the mast cell (MC) numbers within the gut mucosa measured in the ileal small intestinal samples of 3 days post-stroke mice treated with cromolyn compared to the vehicle-treated post-stroke mice. Two-way ANOVA and Turkey’s multiple comparison show the statistical significance among the groups. Data are expressed as mean ± SEM, as well as individual values, and are obtained from > 3 independent experiments at different time points (*n = *6). *P < 0.05, **P < 0.01, ***P < 0.001, ****P < 0.0001. Magnification: 20X

The majority of MC progenitors are housed in the intestinal mucosa [22, 23]. MCs are found elevated after stroke [37]. Oral cromolyn treatment led to a significant reduction in the number of gut MCs within the lamina mucosa and sub-mucosa of mice 3 days after stroke, compared to vehicle-treated stroke mice (*P* < 0.0001) (Fig. 5). Interestingly, cromolyn administration reduced peripheral gut mucosal-associated MC maturation/recruitment to the site of injury at 3 days after stroke. Along with the reduced MCs seen after cromolyn treatment, we also found significantly increased (*P* < 0.0001) mucus 2 (MUC2) positive goblet cells in cromolyn-treated mice compared to vehicle-treated stroke mice at 3 days after stroke (Additional file 3: Figure S3).

### Stroke induced gut dysbiosis was significantly attenuated by oral cromolyn treatment in aged mice 3 days after stroke

**Fig. 6:**
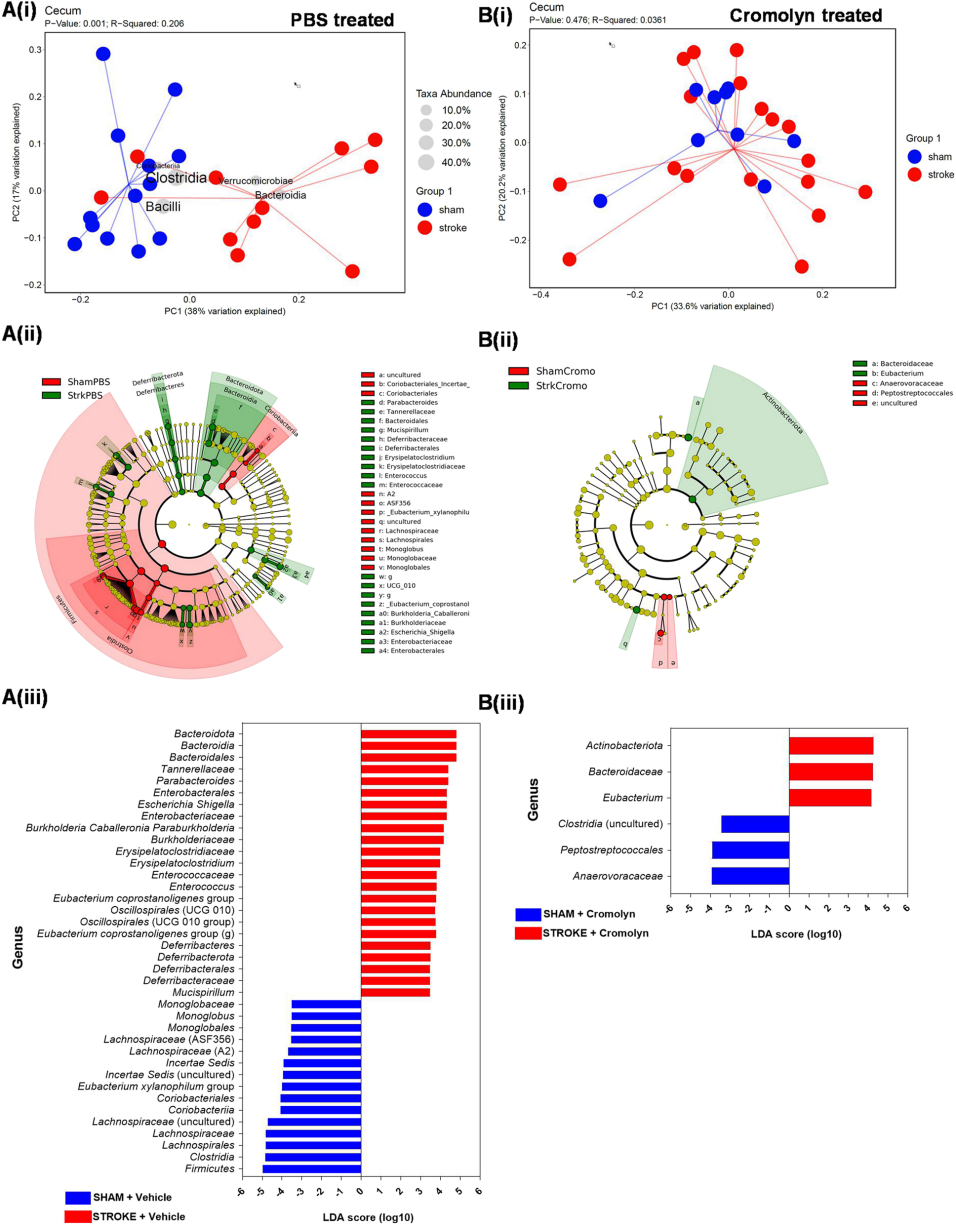
Compositional differences in gut microbiota by 16S rRNA sequencing of intestinal luminal content. **A(i)** Visualization of beta-diversity, or between-samples diversity, with weighted UniFrac distances by principal coordinate analysis (PCoA) shows a clustering effect by strain between mice at 3 days post-stroke compared to age-matched sham controls in PBS vehicle-treated groups (*P = *0.001). **A(ii)** Cladogram showing increased bacterial changes from the phylum to genus levels in 3 days post-stroke animals with vehicle-PBS-treated groups compared to sham groups. **A(iii)** Linear discriminant analysis (LDA) score represents linear combination of features, i.e., family level which separates 2 groups (Groups: Sham-Vehicle compared to Stroke-Vehicle-treated groups for 3 days). Green bars are the family and class level (of the phylogenetic tree) that is upregulated in stroke PBS groups compared to the red bar that are downregulated in Sham PBS groups. **B(i)** Visualization of beta-diversity, or between-samples diversity, with weighted UniFrac distances by principal coordinate analysis (PCoA) shows no effect between mice at 3 days post-stroke compared to age-matched sham controls in cromolyn-treated groups (*P = *0.476). **B(ii)** Cladogram showing no significant changes in bacterial diversity from the phylum to genus levels in 3 days post-stroke animals with cromolyn-treated groups compared to respective sham groups. **B(iii)** Linear discriminant analysis (LDA) score represents linear combination of features, i.e., family level which separates 2 groups (Groups: Sham-cromolyn compared to Stroke-cromolyn-treated groups 3 days post-stroke). Green bars are the family and class level (of the phylogenetic tree) that is upregulated in stroke cromolyn groups compared to the red bar that are downregulated in sham cromolyn groups

Intestinal inflammation can affect the composition of gut microbiota [38]. We performed 16S rRNA sequencing on large intestinal gut contents to examine variations in the microbial diversity caused by stroke-induced inflammation, and determined the effects of cromolyn administration on stroke-induced dysbiosis. There was a significant shift in the beta-diversity or between-samples diversity, with weighted UniFrac distances by principal coordinate analysis (PCoA), at 3 days post-stroke compared to sham controls. As seen in Fig. 6A(i), there is a significant clustering effect (*P = *0.001) along the PC1 axis (38% of the variation explained). Interestingly, when stroke animals were treated with cromolyn, upon visualization of beta-diversity (or between-samples diversity) with weighted UniFrac distances by PCoA at 3 days post-stroke, there was no significant clustering effect (*P = *0.476) along the PC1 axis, compared to the cromolyn-treated sham control group (Fig. 6B(i)). As expected, we did not see significant differences in alpha diversity (measures within sample diversity), and we found no differences in OTUs (*P = *0.7 or 0.5) between the groups (Additional file 4: Figure S4). Additionally, visualization by cladogram shows clear increase in bacterial diversity in stroke and sham animals without cromolyn treatment compared to stroke and sham animals with cromolyn treatment (Fig. 6A(ii), B(ii)). In line with this, the LDA score also clearly shows changes in bacterial families in stroke versus sham animals without cromolyn treatment compared to cromolyn-treated animals (Fig. 6A(iii)). Specific pathobionts (opportunistic pathogens), like Escherichia, Shigella, Enterococcus, Erysipelatoclostridium, were significantly increased, and Clostridium was reduced in vehicle-treated stroke mice. On the other hand, Actinobacteria (a known beneficial bacterial order) was significantly elevated in stroke animals with cromolyn treatment (Fig. 6B(iii)). This shows that cromolyn treatment prevents stroke-induced gut dysbiosis. We found changes in Firmicutes versus Bacteroidetes ratio. Mainly, we found decreased phylum Firmicutes and Bacteroidetes with increased Proteobacteria in vehicle-treated stroke group (*P* < 0.05), which was prevented after cromolyn treatment in aged mice 3 days after stroke.

### MCs migrate into the injured brain from peripheral sources

**Fig. 7:**
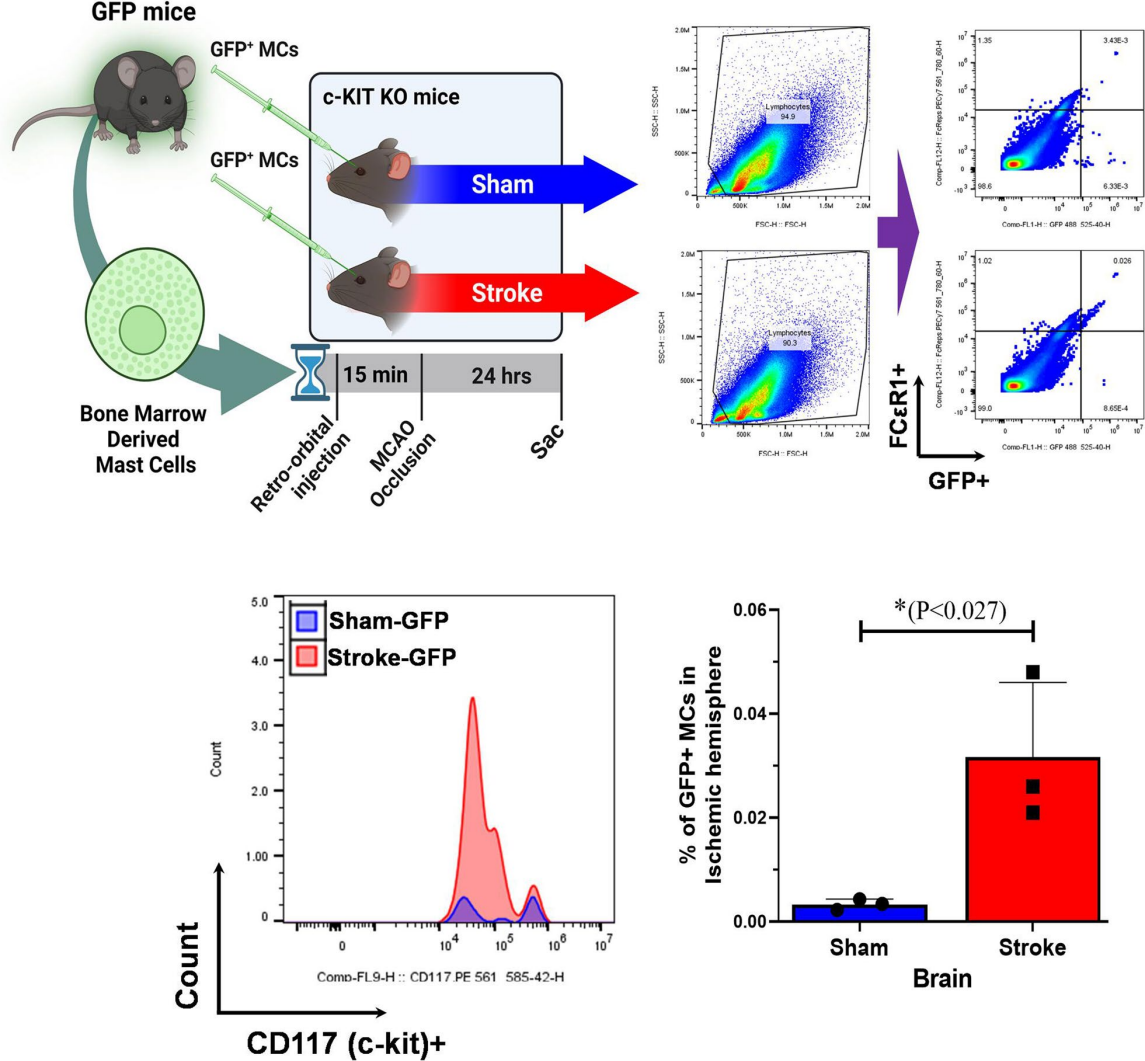
Adoptive transfer of bone marrow-derived GFP + MCs into c-Kitw-sh/cKitw-sh MC knockout (KO) mice caused significant increase in recruitment of systemic MCs to the injured brain 24 h after stroke. Flow cytometry analysis revealed that there is a significant increase in GFP + MCs numbers in the brain of the stroke compared to the sham MC KO mice (*n = *3). Data are expressed as mean ± SEM, as well as individual values. Student’s t-test is used with normally distributed data to analyze statistical significance. *P < 0.05, **P < 0.01, ***P < 0.001

We verified that matured aged MCs migrate and are recruited to the injured brain region after stroke from the peripheral sources through the systemic circulation. To investigate MC migration from peripheral sources into the brain, we used mast cell deficient *c-Kit*^*w−sh*^* /cKit*^*w−sh*^ mice. Middle-aged MC KO mice (12-m old) were injected with 5*10 [6] GFP + bone marrow-derived MCs (BMMC were derived from 10-month-old GFP + WT bone marrow), into the systemic circulation by retroorbital injection 15 min prior to the stroke surgery (Fig. 7). The ischemic hemisphere was harvested and GFP + MCs were quantified 24 h after stroke to investigate the MC migration into the ischemic brain by flow cytometry. BMMC were identified as CD45 + FCεR1 + CD117/c-Kit + GFP + population. We found, in both cases, a significant increase (*P* < 0.027) in the GFP + -BMMC count and as a percentage of GFP + -BMMC in the brain of stroke *c-Kit*^*w−sh*^* /cKit*^*w−sh*^ mice compared to surgical sham *c-Kit*^*w−sh*^* /cKit*^*w−sh*^ mice. This indicates that with brain injury MCs are recruited immediately from peripheral sources during the acute phase of stroke via the systemic circulation.

## Discussion

Stroke is the second leading cause of death and the leading cause of serious long-term disability worldwide [39]. Approximately 800,000 Americans have strokes every year and stroke is the fifth leading cause of death in the US [39]. Over 70% of all strokes occur in people over the age of 65, and most strokes are ischemic [39]. The immune system plays an integral role in the pathogenesis of ischemic stroke and contributes to infarct damage [6]. It is now recognized that aging alters the immunological response to stroke [40]. There is a growing body of evidence that inflammatory cell infiltration is predominantly deleterious in the early phase of ischemia [4]. Mast cells (MCs), best known as first responders and pro-inflammatory effector cells, play critical roles in the development of inflammation in many disease settings [11]. Two types of MCs occur (1) connective tissue MCs and (2) gut mucosal MC progenitors that are derived from the bone marrow. Unlike connective tissue MCs, mucosal MC progenitors are recruited to the site of injury [22, 41]. The pool of committed mucosal MC progenitors in the intestine is greater than that found in bone marrow [23]. We found that stroke elicits a vicious cycle of peripheral inflammation through communication within the brain–gut axis (BGA) [26–28], leading to severe gut dysregulation, which worsens with age. We also found that histamine (HA) release by gut mucosal mast cells (MC) contributes to severe peripheral and central inflammation and immune cell recruitment to the inured brain after stroke, which is enhanced with age [3]. However, if this occurs in patients with stroke, and if stabilizing MC HA release is beneficial in aged mice has not been evaluated previously. In the current study, we show that histamine-loaded mast cells were significantly elevated in the acute and sub-acute phase of stroke, but not at the later chronic time points after stroke in human brains. Interestingly in the later stages of stroke, elevated empty MCs without histamine were seen, suggesting MC activation occurs primarily in the early phase of ischemic injury.

MCs are rapidly recruited to the site of damage (brain) from the gut mucosa after injury. Aged-MCs are known to be in an increased state of activation and have increased HA release [42]. MCs are preferentially located next to nerve terminals in the lamina propria of gut mucosa, where they are activated and release inflammatory mediators [43]. Anatomic connections between MCs and enteric nerve fibers have been demonstrated in human gastrointestinal mucosa and increase with inflammation [44]. This connection provides a physiologic means for bidirectional communication between the “brain and gut” through which ischemic injury can influence gut function and vice versa. We found that MCs maturation in the gut mucosa is significantly increased with stroke and treatment with cromolyn, a known mast cell stabilizer, significantly suppressed MCs maturation in the intestinal mucosa of aged mice at 3 days after stroke.

MCs are the largest repository for histamine (HA) in the body [45]**.** The gut serves as a major source of HA-packed MCs [24]. HA is a biogenic amine [46] that is involved in mast cell maturation [15]. MCs comprise 2–5% of the mononuclear cells in the normal gut mucosa and intestinal inflammation increases this number [43]. HA plays a critical role in various physiological responses, such as inflammation, gastric acid secretion, neurotransmission, and immune modulation, which are mediated by HA binding to its specific GPCR receptors, the HR1, HR2, HR3 and HR4 [15]. Gastrointestinal mucosa predominantly express HR1 and HR2 [46–48]. In the current study, we found significantly elevated HA levels in the circulating blood plasma at 3 days after stroke and this was significantly suppressed after treatment with cromolyn. Cromolyn treatment, initiated at 3 h after stroke inhibited circulating HA levels and significantly decreased gut MC maturation at 3 days post-stroke. HA is known to elevate IL6 and activate MC [33–36, 49] and can influence peripheral inflammation [50]. In our study, we found elevated levels of plasma IL-6 and keratinocytes-derived chemokine (KC) in aged animals 3 days after stroke compared to sham animals, and cromolyn attenuated the stroke-induced elevation in HA, IL-6 and KC.

The mammalian gut harbors a very dense and diverse microbial community, the “intestinal microbiota” (> 10^12^ bacteria /g) which has profound effects on the host [51, 52]. Stable interactions between the host barrier and the intestinal microbiota are required for host homeostasis [51–54]. Gut inflammation can cause gut barrier breakdown (GBB) which in turn leads to “leaky gut” [19–21] characterized by reduced mucus producing goblet cells (first line of defense). Our results show reduced goblet cells with MUC2 in stroke animals compared to sham mice. Cromolyn treatment significantly increased the mucus MUC2-producing goblet cells in the intestine 3 days after stroke compared to vehicle-treated stroke mice. Our previous studies showed significant differences in the bacterial diversity “gut dysbiosis” (pathological imbalance in beneficial bacterial community) after stroke [3, 55, 56]. Reversing the gut dysbiosis with beneficial bacteria via oral supplementation of probiotics and prebiotics improved stroke outcomes in aged mice [56]. Imbalance in gut microbial communities could play a major role in post-stroke inflammation due to leaky gut and the subsequent increase in pathogens in the gut and systemic circulation (known to survive in inflammatory environments) [57]. Probiotics cannot successfully colonize the gut under inflammatory conditions [58]. Therefore, dampening the inflammation followed by subsequent probiotic treatment might have greater therapeutic potential. Since we found reduced pro-inflammatory signals with reduced gut MC maturation after cromolyn treatment post-stroke, we were interested in changes in the gut microbiota diversity after treatment. We found significant gut dysbiosis, with reduced phylum Firmicutes and Bacteroidetes with elevated Proteobacteria at 3 days after stroke in aged mice. These pathological changes have been previously documented and our study was in line with the previous evidences showing elevated Proteobacteria (phylum that contains *Escherichia sps*) [59]. Interestingly, cromolyn administration as early as 3 and 10 h followed by every 24 h for 3 days after stroke had a significant impact on protecting the healthy microbiota and prevented gut dysbiosis post-stroke. We found no changes in the gut microbiota diversity between aged stroke and age-matched sham groups with oral cromolyn treatment. Phylum Actinobacteria (*Bifidobacterium sps*, beneficial probiotics) was elevated with cromolyn treatment 3 days after stroke in the gut. This was associated with increased MUC2 that provides a healthy ecosystem for beneficial microbes to colonize.

Our results demonstrate that aged animals after stroke showed significantly elevated MCs compared to age-matched sham controls and this effect was significantly attenuated after cromolyn treatment. Interestingly, elevated MCs in the brain of naïve aged males compared to young male animals has been seen in previous studies [3]. More importantly, our results showed elevated histamine-filled MCs in the human ischemic brain during the acute and sub-acute phase of stroke, whereas in chronic stroke, MC were devoid of granules suggesting an early response of histamine in the activation of pro-inflammatory signals after stroke. Therefore, treating ischemic stroke patients with a mast cell stabilizer could have translational value, by dampening peripheral and neuroinflammation.

Cromolyn administration significantly improved neurological outcomes measured by NDS score and in left paw positioning by digigait at 3 days after stroke compared to vehicle-treated groups. However, we did not see any changes in the infarct volume, and it is possible that more chronic time points might show a greater effect in cerebral atrophy. Similar findings of behavioral improvement without changes in infarct size have been seen by others, for example, a significant improvement in behavior was seen following treatment with a MEK/ERK inhibitor (U0126) 48 h after ischemia, yet the stroke volume remained unchanged [60].

In the current study, we show that activation of aged gut MCs increases HA-HR signaling and triggers gut mucosal MC activation and elevations in markers of peripheral inflammation. Aged stroke animals treated with an oral MC stabilizer had reduced stroke-induced gut MC activation, reduced gut pathophysiology, less neuroinflammation and improved functional outcomes after stroke. The immune response following stroke is not restricted to the brain, as effects on immune function are also seen in the periphery [6] and these peripheral effects can also contribute to poorer outcomes and increased mortality. New therapies that target peripheral inflammation may protect neural tissue from post-ischemic damage, and reduce mortality and neurological deficits in stroke patients.

## Materials and methods

### Animal experiments

C57BL/6J aged (20 months) male mice and mast cell deficient *c-Kit*^*w−sh*^* /cKit*^*w−sh*^ mice (12 months) were housed in a specific pathogen-free facility (light cycle 12/12 h light/dark). Food and water were provided ad libitum. All animal procedures were performed in accordance with NIH guidelines for the care and use of laboratory animals and approved by the Animal Care Committee of the University of Texas Health Science Center at Houston, McGovern Medical School.

All human tissue samples were obtained from the University of Pittsburgh neurodegenerative brain bank with appropriate ethics committee approval.

### Experimental groups

To examine the mast cells (MC) role in neuroinflammation post-stroke and how they can be stabilized after stroke, we used aged (Ag) mice (18–20 mo). The mice underwent MCAO surgery and they were treated with 25 mg/kg BW cromolyn (mast cell stabilizer) at 3 h, 10 h, 24 h and 48 h post-stroke. They were euthanized at 24 h (*n = *12), and 3 days (*n = *30) post-ischemia; sham mice received the same surgery, but the suture was not inserted into the middle cerebral artery (MCA) (*n = *48). Both groups received both treatments, cromolyn and PBS (Table 1).

**Table 1:** Experimental design in different groups of mice

Group no.	Name of the group	Compound administered by gavage
1	Stroke	Cromolyn (25 mg/kg)
2	Stroke	Vehicle (PBS)
3	Sham (surgery)	Cromolyn (25 mg/kg)
4	Sham (surgery)	Vehicle (PBS)

To explore the idea that matured aged MCs migrate from the periphery, to the injured brain and exacerbate HA mediated neuroinflammation, we used aged mast cell deficient *c-Kit*^*w−sh*^* /cKit*^*w−sh*^ mice (12 months).

### Middle cerebral artery occlusion

Animals in the respective groups underwent transient focal ischemia under isoflurane anesthesia for 60 min by occlusion of the right middle cerebral artery (MCA). Body temperature was maintained at 37.0 ± 1.0 °C throughout the surgery by an automated temperature control feedback loop temperature-controlled heating pad to maintain an internal body temperature of 37 °C during the procedure. Before the initial incision, bupivacaine (0.25%) was administered subcutaneously. Next, the common, external, and internal carotid artery were isolated before a small incision was made in the external carotid in order to insert and guide the 0.23-mm silicone-coated monofilament (6.0) (Doccol, Redlands, CA) into the right internal carotid artery. The mice were allowed to awaken after occlusion and neurological behavioral deficits were evaluated with the neurological deficit score (NDS) test. Animals were re-anaesthetized and the filament was removed to allow for reperfusion after 60 min of occlusion. Post-operative care included daily weighing and 1 ml/kg NaCl subcutaneously and soft show for 7 days. Animals were excluded from the study based on low NDS score and/or confirmed bleeding/hemorrhage during surgery or sac. Animals were placed in a recovery cage. Body weight was recorded daily for the duration of the experiments. All experiments were performed by investigators blinded to animal groups and treatments to reduce experimenter bias (Blinded: Due to the size of these animals and stroke having impact on how the animals behave, we used the following methods to blind our samples and the analysis. We followed the blinding of the harvested samples. Tissue harvesting was performed by a blinded individual and analysis is performed by a separate individual who was also blinded. For IHC, the histology core processed the embedding and sectioning of the samples and was performed blinded. Staining was performed and followed up with imaging and analysis blinded. Only after the quantification, we un-blinded the samples to allocate them to the respective groups).

### Behavioral testing

#### Neurological deficit score test

Neurological deficit score (NDS) was evaluated during occlusion, and 24 h, and 72 h post-reperfusion as previously described [61]. The test is scaled in Table 2

**Table 2:** Neurological deficit scoring system based on specification during occlusion, and 24 h post-reperfusion

Score	Specification
0	No deficit
1	Unilateral torso turning when held by tail
2	Circling when hind limbs are elevated
3	Spontaneous circling
4	Barrel rolling or no spontaneous movement
5	Dead

### Treadmill test/Digigait

Animals were acclimated in the behavior room overnight prior to the start of the experiment. The treadmill test evaluated the mice walking gait and gait symmetry by using the DigiGait system (MouseSpecifics, Inc), which utilizes a transparent treadmill belt and a high-speed camera positioned under the animal capturing the ventral side of the mouse. The treadmill was set to a speed of 10 cm/s, adjusted for aged and stroke impaired mice, and videos were recorded until 10 uninterrupted successive steps were captured. The videos were analyzed using DigiGait Analysis 15 and the gait symmetry was extrapolated from sham-operated, PBS-treated, and cromolyn-treated stroke mice.

### Open field

Motor function and signs of anxious behavior were evaluated through open field testing. The mouse was placed in an open field arena consists of a 40 × 40 × 40 cm box and was allowed to explore it freely for 20 min. Motor function in terms of distance (cm) walked was measured and analyzed in Noldus Ethovision (Noldus Inc., Netherlands). The open field was also divided into a central and boarder section to assess the location of the mouse during the 20-min exploration, with an animal that avoided the center of the arena was deemed to exhibit an anxious behavioral phenotype.

### Tail suspension test

Tail suspension test has been a very useful test in screening antidepressant drugs and assessing depressive behavior in mouse behavior. During the test, mice were suspended by their tail approximately 15 cm above the table surface for 6 min. During this time, they were recorded and their escape-oriented behavior was quantified by manual review of the recordings. The setup allowed for two mice to be suspended at the same time, separated by a screen so that they could not see or interact with each other. Small, 1.5-cm plastic cylinders, 1-cm diameter, were fashioned and placed on the base of their tails in order to prevent the mice from successfully climb their tails, as the C57BL/6 mice are notorious for climbing during the test [62]. Depressive behavior was measured in terms of lack of escape-oriented behavior, immobility, and plotted as a percentage of the total 6-min time period.

### Infarct volume

Brains were sectioned, on ice, into 2-mm-thick sections and stained with 2.5% triphenyl tetrazolium chloride (TTC) (1) dye for 20 min at room temperature to allow for a quick assessment of ischemic damage. Once TTC staining was complete, the brain sections were stored in 4% PFA overnight. Finally, the sections were placed in PBS at 8 °C until quantification was performed. Infarct volumes were analyzed using the Sigma Scan Pro 5 software as previously described. Total volumes of the contralateral and ipsilateral hemisphere, including cortex and striatum, were measured and the final infarct volumes are presented as a percentage of the contralateral structure (Swanson’s method to correct for edema).

### Immunohistochemistry

Formalin-fixed, paraffin-embedded intestinal (cecal, colon and ileum) tissue section (4 μm) were incubated overnight at 4 °C with a primary antibody (Table 3) after antigen retrieval according to the manufacturer’s instructions.

**Table 3:** Information of antibodies

Name of antibody	Company name and Catalog no.	Dilution factor
Anti-Mast Cell Tryptase antibody [AA1]	abcam, ab2378	1:1000
Anti-IL-6 antibody [MP5-20F3]	abcam, ab191194	1:100
MUC2 Antibody	Novus, 31231	1:100
Goat Anti-Rabbit IgG H&L (HRP)	abcam, ab205718	1:1000
Goat Anti-Mouse IgG H&L (HRP)	abcam ab97023	1:2500
Recombinant Human GDF11 protein (ab50159)	abcam ab50159	1:1000

Samples were washed and subsequently incubated with secondary antibody for 45 min at RT (Table 3) Sections were counterstained either with the diaminobenzidine substrate kit (Nacalai, USA) followed by hematoxylin, or sections were stained with 6-diamidino-2-phenylindole (DAPI, Thermo Fisher, USA) for visualization of cell nuclei as previously described.

### Microscopy

Images were obtained on a Keyence BZ-X810 all in one confocal microscope at 20 ×, 40 × and 60 × magnification. Images were processed for brightness and contrast correction, cropping, and addition of scale bars with Keyence BZ-X800 Analyzer 1.1.1.8. software.

### Toluidine blue staining in human autopsy brain

Formalin-fixed paraffin-embedded human brain autopsy sections were cut at 30 μm. The slides were deparaffinized and hydrated. Slides were treated with toluidine blue working solution (1% toluidine blue ethanol solution in 1% sodium chloride) followed by dehydration. Nuclei were counterstained with hematoxylin. Human brain autopsy samples were obtained from stroke patients (acute, sub-acute and late sub-acute to old post-stroke and controls). The infarct region is from the cortex. MCs were quantified by counting the positively stained cells around the infarct region per section with a 5 ×, 20 × and 40 × magnification.

### Intestinal content collection and 16S rRNA gene sequencing

Microbiota in the intestinal luminal content (ileum, cecum and feces) samples were collected from mice at the time of tissue harvest and stored in sterile tubes at − 80 °C until analyzed. The bacteria taxa in each sample were analyzed by amplifying the V4 variable region of the 16S ribosomal RNA (rRNA) gene using high-throughput sequence analysis (Illumina MisSeq platform; Illumina, San Diego, CA) [63]. Quality filtered 16S rRNA sequences were clustered into operational taxonomic units (OTUs), with 97% similarity, by closed reference OTU-picking using the UCLUST algorithm and GreenGenes reference database (v13.5) as implemented in Quantitative Insights into Microbial Ecology (QIIME versions 1.6 and 1.7) [64]. Sequences were checked for chimeras using ChimeraSlayer with standard options as implemented in QIIME. Sequences not clustered were identified using the Ribosomal Database Project to the lowest possible taxonomic level [65]. The data were randomly rarefied to 10,000 sequences per sample before any downstream analysis. ATIMA (Agile Toolkit for Incisive Microbial Analyses) developed by Alkek Center for Metagenomics and Microbiome Research (CMMR) at Baylor College of Medicine was used for analysis and visualization of microbiome data sets.

### Linear discriminate analysis effect size (LEFSe)

LEFSe and plot cladogram analysis was performed through the Huttenhower lab galaxy site (https://huttenhower.sph.harvard.edu/galaxy) using subsampled 16S rRNA gene sequence data (without prescreening) isolated from cecal samples (described above). The LEFSe algorithm was used to identify taxa characterizing the differences between 2 groups [66].

### mRNA gene expression in the intestinal mucosa

To quantify relative mRNA expression levels of interferon (IFN)-γ, tumor necrosis factor (TNF)-α, interleukin (IL)-6 mRNA was extracted from intestinal mucosa samples (ileum and cecum) using the miRNeasy^®^ mini kit (QIAGEN).

One microgram of RNA was reverse-transcribed to single stranded cDNA using the RevertAid H minus First Strand cDNA Synthesis Kit (Thermo Fisher, USA). Reverse transcriptase real-time (RT) PCR was performed using the Quant Studio 3 Real-Time PCR system (Applied Biosystems, USA). The RT-PCR reaction mix (adjusted with H2O to a total volume of 20 μl) contained 1 μl template DNA, 10 μl Power SYBR Green PCR master mix (ABI), and 0.5 μl of the respective primers (10 μM each). The forward and reverse primers used for IFN-γ, TNF-α, and IL-6 quantification were described previously [19, 46].

Relative mRNA target gene expression levels (Ratio* = *[(Etarget) dCPtarget (control-sample)]/[(Eref.) dCPref. (control- sample)]) were normalized to the house keeping gene glyceraldehyde 3-phosphate dehydrogenase (GAPDH) and used as a reference.

Intestinal mucosal cytokine of the sham-saline control group was set to 1.0 and used as the calibrator to identify the relative mRNA fold difference between the cromolyn and saline-treated sham and stroke groups.

### Bone marrow-derived GFP-mast cell (BMMC) maturation

Bone marrow (BM) cells will be derived from 10-m-old GFP-transgenic C57BL/6-TgN mice. BM will be stimulated under the laboratory conditions as described [67]. Briefly, the BM cell culture was established at a density of 3 × 10^6^ cells/ml in IMDM, supplemented with 10% FCS (inactivated at 56 °C), 2 mM l-glutamine, 1 mM pyruvate, 100 U/ml penicillin, 100 μg/ml streptomycin, 50 U/ml IL-4 (20 ng/ml), and 20 U/ml mIL-3 (15 ng/ml). Non-adherent cells were transferred to fresh medium every 2–3 days for a total of at least 23 days to remove adherent macrophages and fibroblasts. The matured GFP^+^ MCs granule formation will be analyzed by Toluidine blue and administered to MC-deficient mice.

### Adoptive transfer

Donor cells were obtained from BMMC GFP + MC cultures and would a have sufficient number of cells were cultures for transfer. GFP + MCs (10^6^ cells) were resuspended in 37 °C PBS and retro-orbitally injected into the anesthetized c-Kit KO recipient mice using a BD insulin syringe with the BD Ultra-Fine needle (12.7 mm × 30-gauge, 3/10 ml/cc). Animals were left untouched for 15 min and were followed by MCAO occlusion or sham surgery. Animals were killed 24 h post-stroke. Brain ischemic hemispheres were collected and flow cytometry was performed.

### Flow cytometry

Brain: After removal of intestinal tissue, mice were transcardially perfused with 60 ml cold, sterile PBS prior to aseptic removal of the spleen, lung, and brain tissues. Brain tissue was placed in complete Roswell Park Memorial Institute medium 1640 (Lonza) medium and mechanically and enzymatically digested in Collagenase/Dispase (1 mg/ml) and DNase (10 mg/ml; Roche Diagnostics) for 45 min at 37 °C. The cell suspension was filtered through a 70 μm filter. Leukocytes were harvested from the interphase of a 70–30% and 70–40% Percoll gradients for the brain. MCs were gated for FCεR1 + with GFP + and for CD45 positive (+) followed by FCεR1 + with CD117 (c-Kit +) expression and as well.

Flow cytometry in c-KIT KO mice experiment, cells were gated for GFP + (MCs that were adoptive transferred) and FcεR1 + cells, followed by cell count of c-Kit expressing cells between sham and stroke c-Kit knock-out mice.

### Cresyl violet staining

Brains were harvested after the mice were perfused transcardially using icecold PBS solution. Then they were collected and add into a 4% paraformaldehyde solution to fix the tissue, followed by a cryoprotection in 30% sucrose solution for 72 h. Each brain was embedded in TFM embedding solution and frozen at − 80 °C for 24 h, then sectioned (12 µm) in a coronal plane using a cryostat at − 25 °C. Sections were mounted on charged slides stained with cresyl violet and imaged for further analyzing of infarct volumes and evaluation of ischemic cell damage [68, 69].

### Mass spectrometry histamine quantification

Blood was collected by cardiac puncture before perfusion and centrifuged (14,000 g for 15 min at 4 °C) and plasma was collected and stored frozen (at − 80 °C) until use. Changes in circulating histamine were examined by mass spectrometry. Histamine concentrations were quantified in the blood plasma for stroke and sham groups both treated with cromolyn and saline solution. Blood plasma was processed through methanol (Sigma-Aldrich, USA) separation. The obtained supernatants were transferred into 3-kDa filtrate and centrifuged for 14,000 g, 40 min at room temperature. Flow-through was collected, and mass spectrometry analysis was performed. Mass spectrometric quantification was performed as follows.

Histamine, formic acid (FA), and perfluoroheptanoic acid (PFHA) were obtained from Sigma-Aldrich (St. Louis, MO). Histamine-α,α,β,β-d4 was obtained from CDN isotopes (Pointe-Claire, Canada). Water and acetonitrile (ACN) were obtained from Fisher Scientific (Waltham, MA). The histamine-d4 internal standard solution was prepared at a concentration of 250 ng/ml of d4-histamine in water with 0.1% FA. Thirty microliters of internal standard solution were added to 30 μL of each sample, vortexed for 1 min, and dried in a SpeedVac for 5 h. Thirty microliters of water: 0.1% FA was added to each sample, vortexed for 1 min and centrifuged for 5 min at 10,000 rpm. Samples were then loaded into 0.5 ml autosampler vials for quantification. Chromatography was performed on a Shimadzu (Kyoto, Japan) Nexera-XR HPLC system consisting of a SIL-20ACxr autosampler, a CTO-20 AC column oven, and 2 LC-20ADxr binary pumps. Samples were loaded onto a Phenomenex (Torrance, CA) 1 mm × 50 mm phenylhexyl reversed-phased column equipped with a Phenomenex phenylhexyl 4 mm × 2 mm guard column. The aqueous mobile phase (A) consisted of H2O:ACN: FA:PFHA (99.3:0.5:0.1:0.1 v/v/v/v), and the organic mobile phase (B) consisted of H2O:FA (99.9:0.1 v/v). Column flow was 80 μL/min, and 5 μL of sample was injected onto the column and eluted with a constant mobile phase flow rate of 80 μL/min. The elution gradient was optimized as follows: started from 10% B and increased to 70% B over 5 min; ramp to 80% B for 6 s and held for 1 min; ramp back to 10% B over 6 s and maintained at 10% for a total chromatographic run time of 12 min to re-equilibrate.

Selected reaction monitoring (SRM) was performed on a Sciex (Framingham, MA) 6500 QTRAP with a Turbo V source. The mass spectrometer was operated in the positive ion mode under the following conditions: curtain gas 20 psi; collision gas: HIGH; spray voltage 4.5 kV; ion source gas 1 20 psi; ion source gas 2 20 psi; interface heater temperature 175 °C; Q1 and Q3 resolution: unit; scan time 100 mS; declustering potential 100 V; entrance potential 8 V; collision exit potential 10 V. The instrument was calibrated by using Sciex PPG calibration standard and tuned to the manufacturer’s specifications. SRM transitions monitored for histamine were 112 → 95 (20 eV) and 112 → 68 (30 eV). For histamine-d4, the SRM transitions 116 → 99 (20 eV) and 116 → 72 (30 eV) were monitored. Data were acquired with the Analyst^®^ Software (ver 1.6.2) and quantification performed using the Multiquant™ Software (ver 3.0.1).

### Statistics

Values are presented as mean ± standard error for normally distributed data and median with range for not-normally distributed data. Statistical significance was set at *P* < 0.05. Parametric analysis consisted of a Student’s *t*-test or two-way analysis of variance (ANOVA) followed by Holm–Sidak, Bonferroni, or Tukey post hoc analysis, when appropriate. Because the NDS consists of categorical data, the Kruskal–Wallis ANOVA on ranks was used followed by a Dunn’s post hoc analysis, when appropriate. The Mantel–Cox and the Gehan–Breslow–Wilcoxon tests were used to analyze survival after MCAO. Differences in Phyla in the gut microbiota of young and aged mice were analyzed using the unweighted UniFrac distance and plotted in a principal coordinate analysis (PCoA). The UniFrac distance is a measure that considers the branch length shared by the young and aged microbiota when placed on a common phylogenetic tree. All statistical analyses were performed with GraphPad Prism 9. Simple randomization has been applied to experimental groups during experimental design and samples were blinded for all the histological analysis and behavioral studies.

### Supplementary Information

**Additional file 1: Figure S1.** Open field test was performed on mice 3 days post MCAO. A) Total distance moved during the 20-min time period showed no differences between the groups in general motor function. B) The time spent in the center vs border of the arena is an indicator of anxious behavior. Less time spent in the center suggest a more anxious behavioral phenotype. C) The % of time spent in the center of the arena. The high % spent in the center of the arena is directly linked to the relatively low mobility of the mice suggesting that due to, age related low mobility, as mice are placed in the center of the arena at the start of the test. Two-way ANOVA and Turkey’s multiple comparison show the statistical significance among the groups. *P < 0.05, **P < 0.01, ***P < 0.001.

**Additional file 2: Figure S2.** Plasma cytokines measured from human blood plasma of stroke patients. (A) Increased levels of plasma G-CSF at 12 h, 24 h and 72 h after stroke, (B) plasma levels of IL-1R and (C) IL-6 at 12 h, 24 h and 72 h after stroke with aged matched controls. Data are expressed as mean ± SEM. Two-way ANOVA and Turkey’s multiple comparison show the statistical significance among the groups. *P < 0.05, **P < 0.01, ***P < 0.001.

**Additional file 3: Figure S3.** Immunohistochemistry staining followed by quantification of goblet cells in the ileal tissue of mice. Significant increase in the goblet cell numbers within the gut mucosa measured in the ileal small intestinal samples of 3 days post-stroke mice treated with cromolyn compared to the vehicle-treated post-stroke mice and sham controls. Two-way ANOVA and Turkey’s multiple comparison show the statistical significance among the groups. Data are expressed as mean ± SEM, as well as individual values, and are obtained from > 3 independent experiments at different time points. (*n = *6). *P < 0.05, **P < 0.01, ***P < 0.001, ****P < 0.0001. Magnification: 20X.

**Additional file 4: Figure S4.** Visualization of alpha diversity. Bacterial compositional differences within sample diversity in gut microbiota by 16S rRNA sequencing of intestinal luminal content obtained from mice 3 days post MCAO.

**Additional file 5.** Human Brain Autopsy sections from Stroke and Healthy control samples.

**Additional file 6.** Human blood plasma from acute to chronic stroke patients and healthy control subjects.

## Data Availability

Necessary materials and information about human sample data sets are provided as Additional files 5, 6. Bacterial sequencing data set will be made available upon request.
