# Supplementary figures and images for: Stabilizing histamine release in gut mast cells mitigates peripheral and central inflammation after stroke
Source: J Neuroinflammation. 2023 Oct 7;20:230. doi: 10.1186/s12974-023-02887-7 (PMC10560441; doi:10.1186/s12974-023-02887-7)

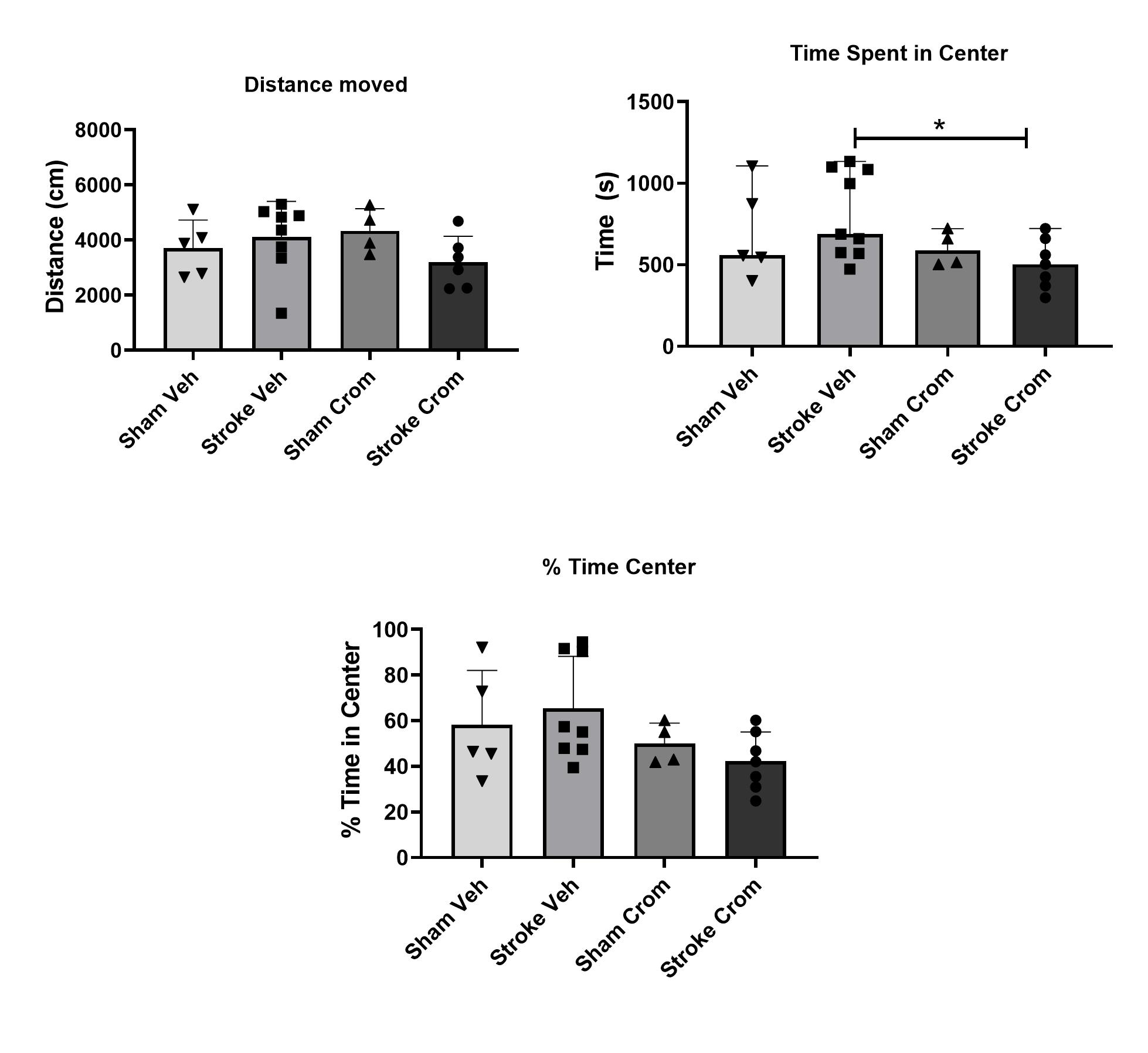

Supplement: Supplementary file 1 — Additional file 1: Figure S1. Open field test was performed on mice 3 days post MCAO. A) Total distance moved during the 20-min time period showed no differences between the groups in general motor function. B) The time spent in the center vs border of the arena is an indicator of anxious behavior. Less time spent in the center suggest a more anxious behavioral phenotype. C) The % of time spent in the center of the arena. The high % spent in the center of the arena is directly linked to the relatively low mobility of the mice suggesting that due to, age related low mobility, as mice are placed in the center of the arena at the start of the test. Two-way ANOVA and Turkey’s multiple comparison show the statistical significance among the groups. *P < 0.05, **P < 0.01, ***P < 0.001. [file 12974_2023_2887_MOESM1_ESM.jpg]

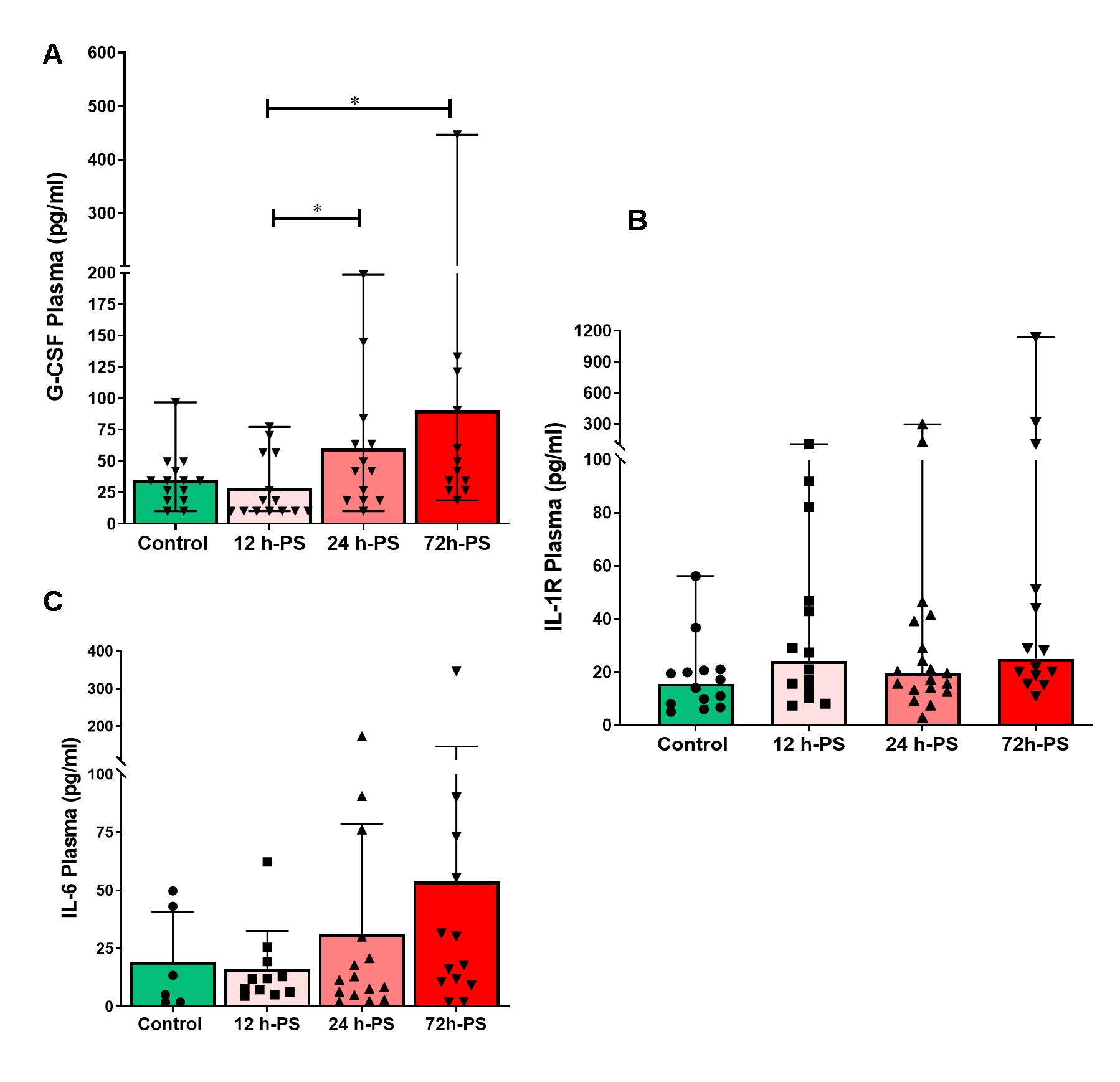

Supplement: Supplementary file 2 — Additional file 2: Figure S2. Plasma cytokines measured from human blood plasma of stroke patients. (A) Increased levels of plasma G-CSF at 12 h, 24 h and 72 h after stroke, (B) plasma levels of IL-1R and (C) IL-6 at 12 h, 24 h and 72 h after stroke with aged matched controls. Data are expressed as mean ± SEM. Two-way ANOVA and Turkey’s multiple comparison show the statistical significance among the groups. *P < 0.05, **P < 0.01, ***P < 0.001. [file 12974_2023_2887_MOESM2_ESM.jpg]

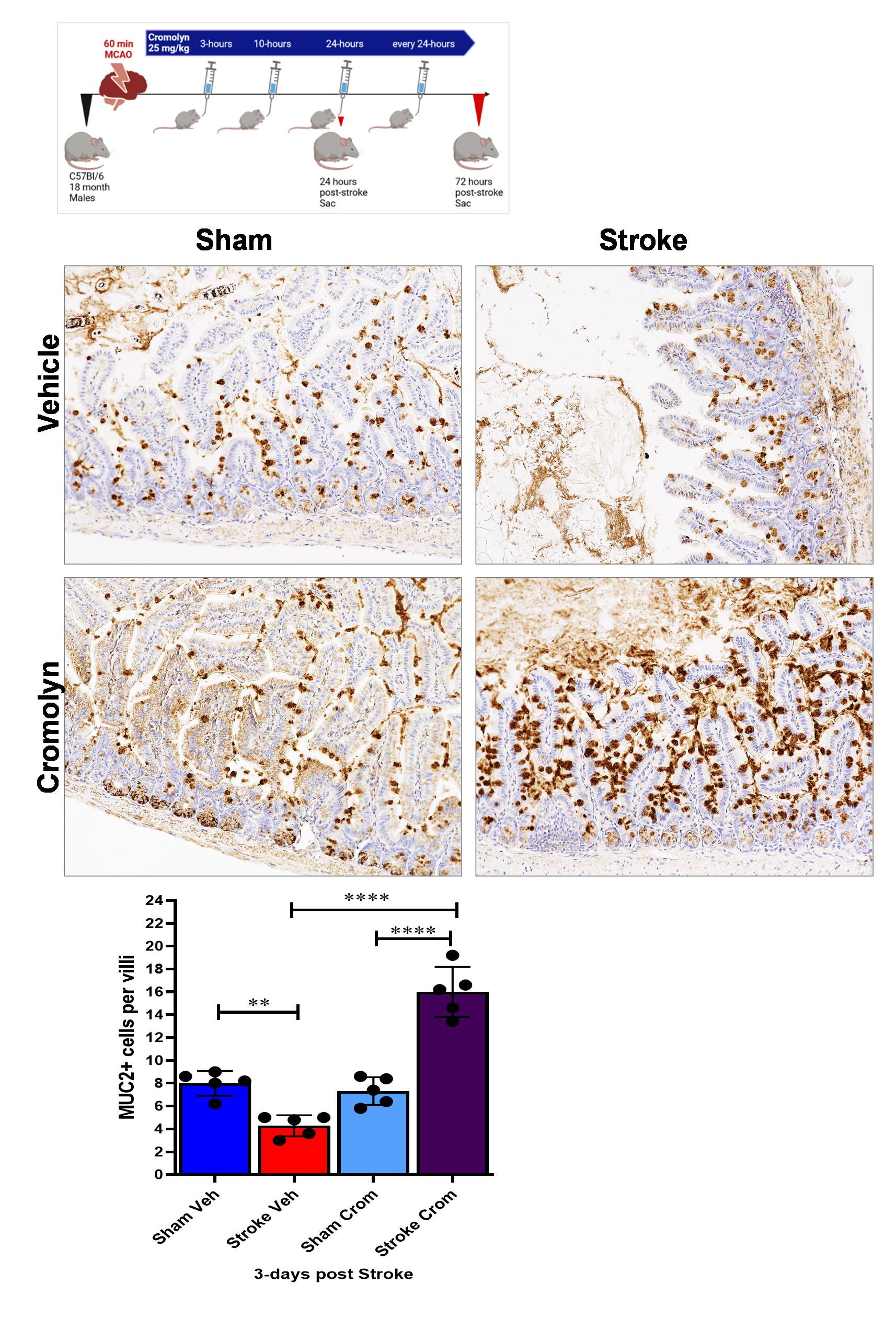

Supplement: Supplementary file 3 — Additional file 3: Figure S3. Immunohistochemistry staining followed by quantification of goblet cells in the ileal tissue of mice. Significant increase in the goblet cell numbers within the gut mucosa measured in the ileal small intestinal samples of 3 days post-stroke mice treated with cromolyn compared to the vehicle-treated post-stroke mice and sham controls. Two-way ANOVA and Turkey’s multiple comparison show the statistical significance among the groups. Data are expressed as mean ± SEM, as well as individual values, and are obtained from > 3 independent experiments at different time points. (n = 6). *P < 0.05, **P < 0.01, ***P < 0.001, ****P < 0.0001. Magnification: 20X. [file 12974_2023_2887_MOESM3_ESM.jpg]

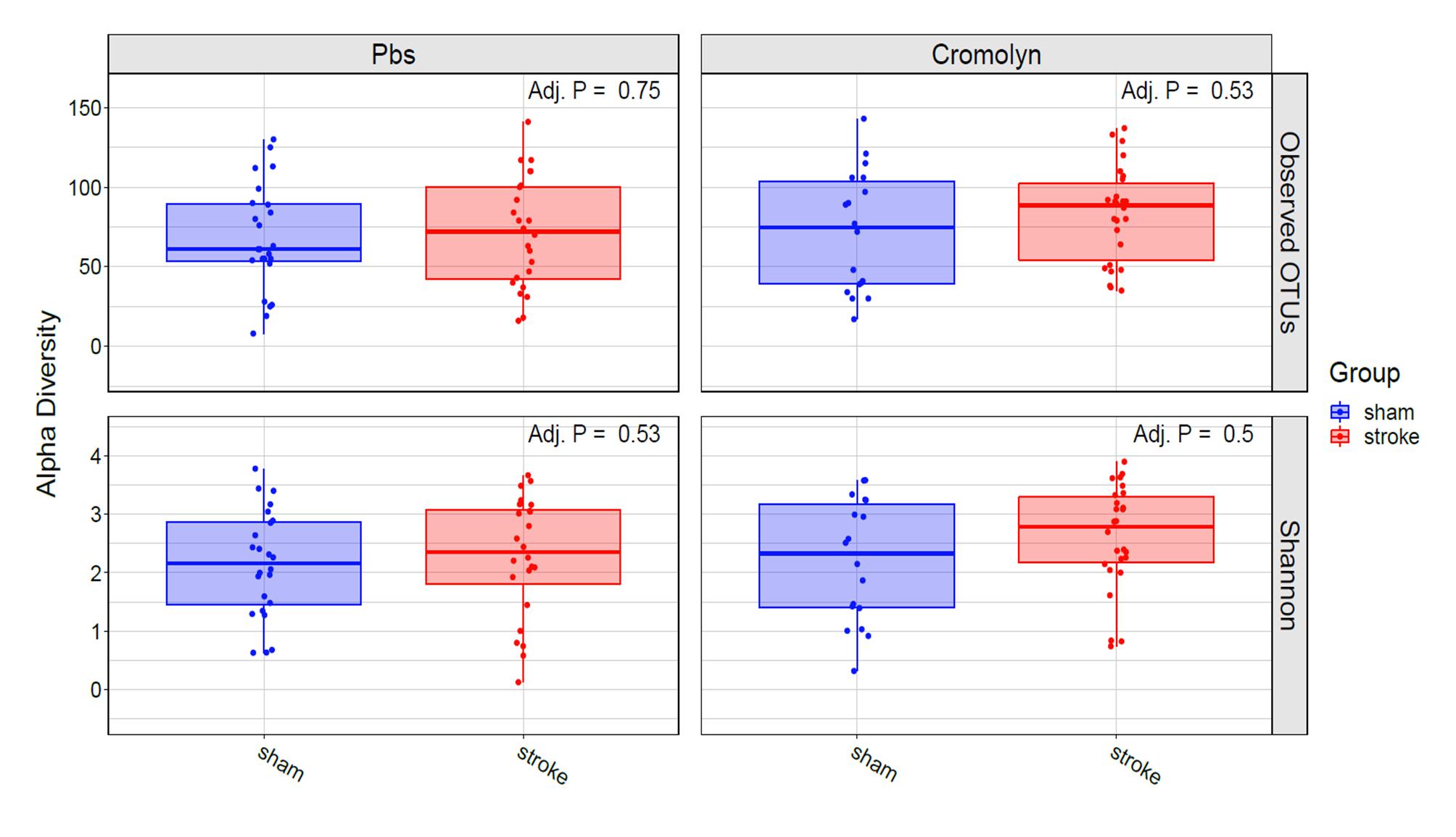

Supplement: Supplementary file 4 — Additional file 4: Figure S4. Visualization of alpha diversity. Bacterial compositional differences within sample diversity in gut microbiota by 16S rRNA sequencing of intestinal luminal content obtained from mice 3 days post MCAO. [file 12974_2023_2887_MOESM4_ESM.jpg]
